# Advancements in elucidating the pathogenesis of actinic keratosis: present state and future prospects

**DOI:** 10.3389/fmed.2024.1330491

**Published:** 2024-03-19

**Authors:** Zhongzhi Wang, Xiaolie Wang, Yuanyang Shi, Siyu Wu, Yu Ding, Guotai Yao, Jianghan Chen

**Affiliations:** ^1^Department of Dermatology, Shanghai Fourth People’s Hospital, Tongji University, Shanghai, China; ^2^Department of Dermatology, Changzheng Hospital, Naval Medical University, Shanghai, China; ^3^Department of Dermatology, Naval Medical Center, Naval Medical University, Shanghai, China

**Keywords:** actinic keratosis, mechanisms, malignant transformation, human papillomavirus, UV

## Abstract

Solar keratosis, also known as actinic keratosis (AK), is becoming increasingly prevalent. It is a benign tumor that develops in the epidermis. Individuals with AK typically exhibit irregular, red, scaly bumps or patches as a result of prolonged exposure to UV rays. These growths primarily appear on sun-exposed areas of the skin such as the face, scalp, and hands. Presently, dermatologists are actively studying AK due to its rising incidence rate in the United States. However, the underlying causes of AK remain poorly understood. Previous research has indicated that the onset of AK involves various mechanisms including UV ray-induced inflammation, oxidative stress, complex mutagenesis, resulting immunosuppression, inhibited apoptosis, dysregulated cell cycle, altered cell proliferation, tissue remodeling, and human papillomavirus (HPV) infection. AK can develop in three ways: spontaneous regression, persistence, or progression into invasive cutaneous squamous cell carcinoma (cSCC). Multiple risk factors and diverse signaling pathways collectively contribute to its complex pathogenesis. To mitigate the risk of cancerous changes associated with long-term UV radiation exposure, prompt identification, management, and prevention of AK are crucial. The objective of this review is to elucidate the primary mechanisms underlying AK malignancy and identify potential treatment targets for dermatologists in clinical settings.

## 1 Introduction

AK, is a harmless growth within the epidermis that is becoming more common nowadays. The main presentation is characterized by atypical epidermal keratinocyte proliferation and chronic UV radiation resulting in red, scaly papules or plaques. It is well recorded that AK is frequently present on areas of skin that are exposed to the sun, including the face, scalp that is balding, and the back of the hands, particularly in older males with fair skin (1). AK is among the most frequently assessed skin disorders by dermatologists, with an estimated incidence of nearly 40 million and an annual cost exceeding $1 billion (USD) in the USA in 2004. The causes of AK are still unknown, and less attention is given to AK compared to other types of skin cancer (2). Previous studies have shown that AK can develop in three ways: spontaneous resolution, persistence, or progression to invasive cSCC. Its complex pathogenesis involves multiple risk factors and diverse signaling pathways. Therefore, it is crucial to promptly diagnose, treat, and prevent AK in order to reduce the risk of developing cSCC due to chronic UV radiation exposure (2). Extensive research has been conducted to investigate various mechanisms and pathways associated with AK signaling. The purpose of this assessment is to elucidate the seven primary processes of AK leading to cancerous transformation. Its aim is to mitigate the risk of malignant transformation and provide clinical dermatologists with potential treatment objectives.

The objective of this analysis was to examine recent research and gain a comprehensive understanding of the main causes of AK. Furthermore, the review highlighted the limitations of previously conducted studies, offering valuable insights for future research directions.

## 2 UV radiation exposure

The primary cause of AK is the accumulation of excessive ultraviolet radiation from the sun. This excessive UV radiation can disrupt complex regulatory pathways involved in cell growth and differentiation, leading to various pathological alterations in epidermal keratinocytes. AK is formed when dysplastic intra-epidermal keratinocytes proliferate, and this proliferation is enhanced by factors such as DNA damage, inflammation, immunosuppression, and mutagenesis. Additionally, exposure to UV radiation stimulates the production of arachidonic acid, pro-inflammatory cytokines, adhesion molecules, and mediators derived from mast cells (1, 3, 4). In addition, being exposed to UV radiation can function as a promoter of tumors, starting events that lead to cancer, resulting in changes to genes, and even hastening the advancement of AK to cSCC (5). AK seems to exhibit an intermediate level of mutational burden compared to both normal skin that is habitually exposed to sunlight (photodamaged skin) and cSCC (6). The alteration of UV is not related just to epidermal keratinocytes but also to fibroblasts, the suppression of the Notch effector CSL (also referred to as RBP-Jj) in dermal fibroblasts alone is adequate to trigger the activation of cancer-associated fibroblasts (CAFs) and subsequently lead to the development of tumors derived from keratinocytes. This phenomenon has been observed in stromal fibroblasts present in premalignant AK lesions as well as *in situ* SCCs (7). Numerous studies have suggested that canonical DNA bases poorly absorb UVA radiation (315–400 nm), leading to indirect DNA damage. Moreover, exposure to UVA radiation results in the production of reactive oxygen species (ROS) like superoxide anions, hydroxyl radicals, and hydrogen peroxide, which initiate oxidative harm in nucleic acids, membrane lipids, and proteins. Abnormal cell growth can be caused by the impairment of regular pathways for transmitting cellular signals. Additionally, UVA radiation causes the creation of 8-hydroxyguanine adducts, resulting in characteristic changes from thymine (T) to guanine (G) known as signature transitions (4). On the other hand, DNA can easily absorb UVB radiation (280–315 nm) and result in direct damage to DNA. Recent research has shown that UVB radiation causes cytosine-containing cyclobutane pyrimidine dimers and pyrimidine-pyrimidone 6–4 photoproducts, leading to C- > T and CC- > TT signature transitions that significantly interfere with normal replication and transcription processes. In a study conducted on mice, both continuous and intermittent regimens of chronic UVB treatment resulted in the development of skin tumors in all cases, demonstrating a 100% incidence rate. While the progression of this process was delayed upon discontinuation of chronic UVB exposure intermittently, it was not entirely prevented. This suggests that prolonged avoidance of UVB exposure merely postpones the timeline of tumor development (8). Moreover, c-Jun N-terminal Kinases (JNKs) and p38 Mitogen-Activated Protein Kinases (MAPKs) are activated by environmental stresses, pro-inflammatory cytokines, DNA damage, and oxidative stress, leading to the initiation of various intracellular signaling pathways such as stress adaptation, proliferation, differentiation, and apoptosis. Additionally, p38 MAPK serves as the precursor kinase for Mitogen and Stress Activated protein Kinase (MSK1) and phosphorylated H2AX (γ-H2AX), both of which play a role in the development of UVB-induced skin cancer (4). UV damage hastens the accumulation of mutations. With minimal damage, the skin’s appearance typically remains normal for most individuals. However, as damage progresses, individuals with fair skin typically demonstrate heightened sensitivity to ultraviolet radiation due to lower melanin content in their skin. Consequently, when exposed to sunlight, fair-skinned individuals are more prone to photodamage, thereby elevating the risk of developing AK. The rate of malignant transformation to SCC per individual AK lesion per year has been estimated to range from 0.025 to 20%, usually falling below 1%. However, up to 60% of SCCs originate from pre-existing AKs, justifying the need for therapy (10, 11). The events mentioned above concurrently contribute to the acceleration of AK’s progression to cSCC (Table 1).

**Table 1:** Common mutated genes of AK.

Genes	Molecular function	Biological process	Expression	References
TP53	Activator, DNA-binding, Repressor	Apoptosis, Biological rhythms, Cell cycle, Host-virus interaction, Necrosis, Transcription, Transcription regulation	Up	Jacobs et al. (12)
MYC	Activator, DNA-binding	Transcription, Transcription regulation	Up	Toll et al. (13)
NOTCH	Activator, Developmental protein, Receptor	Angiogenesis, Differentiation, Notch signaling pathway, Transcription, Transcription regulation	Up	South et al. (14)
RAS	Cell proliferation, Cell differentiation	Adenylate cyclase-activating G protein-coupled, Receptor signaling pathway, Positive regulation of adenylate cyclase activity, Protein localization to bud neck	Up	Corchado-Cobos et al. (15)
RB1	Chromatin regulator, DNA-binding, Repressor	Cell cycle, Host-virus interaction, Transcription, Transcription regulation	Up	Murao et al. (16)
CDKN2A	DNA-binding	Apoptosis, Cell cycle, rRNA processing, Transcription, Transcription regulation, Ubl conjugation pathway	Up	Pickering et al. (17)
FBXW7	Ubiquitination degradation	Biological rhythms, Host-virus interaction, Ubl conjugation pathway	Up	Kim et al. (18)
PIK3CA	Kinase, Serine/threonine-protein kinase, Transferase	Angiogenesis, Lipid metabolism, Phagocytosis	Up	Kim et al. (18)
ASXL1	Chromatin regulator, Repressor	Transcription, Transcription regulation, Ubl conjugation pathway	Up	Kim et al. (18)
FGFR3	Kinase, Receptor, Transferase, Tyrosine-protein kinase	Apoptosis	Up	Kim et al. (18)
EGFR	Developmental protein, Host cell receptor for virus entry, Kinase, Receptor, Transferase, Tyrosine-protein kinase	Host-virus interaction	Up	Murao et al. (16)
EZH2	Chromatin regulator, Methyltransferase, Repressor, Transferase	Biological rhythms, Transcription, Transcription regulation	Up	Kim et al. (18)
IRF4	Activator, DNA-binding	Transcription, Transcription regulation	Up	Jacobs et al. (12)
MC1R	G-protein coupled receptor, Receptor, Transducer	Apoptosis, Notch signaling pathway	Up	Jacobs et al. (12)
TYR	Monooxygenase, Oxidoreductase, Tumor antigen	Melanin biosynthesis	Up	Jacobs et al. (12)
APC	Signal transduction	Wnt signaling pathway	Down	Wang et al. (19)
MSH2	DNA-binding	DNA damage, DNA repair	Down	Sun et al. (20)
MLH1	DNA-binding	Cell cycle, DNA damage, DNA repair	Down	Kim et al. (18)
CDH1	cell–cell adhesions, mobility and proliferation	Cell adhesion	Down	Murao et al. (16)
TGF-b	cell proliferation	Transcription, Transcription regulation	Down	Thomson et al. (21)
SMAD2	DNA-binding	Transcription, Transcription regulation	Down	Xu et al. (22)
SMAD4	DNA-binding	Transcription, Transcription regulation	Down	Xu et al. (22)
CHK1	Kinase, Serine/threonine-protein kinase, Transferase	Cell cycle, DNA damage, DNA repair	–	Ming et al. (23)
KLF4	Activator, DNA-binding	Transcription, Transcription regulation	–	Lu et al. (24)
PTEN	Hydrolase, Protein phosphatase	Apoptosis, Lipid metabolism, Neurogenesis	–	Ming et al. (23)

## 3 Inflammation

The development of squamous cell carcinoma is strongly associated with chronic inflammation. This inflammation can be caused by inflammatory diseases such as AK resulting from exposure to ultraviolet radiation. The inflammatory state activates signals like nuclear factor kappa B (NF-kB) and mitogen-activated protein kinases (MAPKs), which encourage tumor growth. It also leads to the release of pro-inflammatory cytokines, prostaglandins, and reactive oxygen species (ROS). Inflammation and cancer development are closely linked to pathological processes influenced by inflammasomes, autophagy, and sirtuins (12). Recent research suggests that up to 25% of identified tumors have a significant inflammatory component, highlighting the substantial impact of persistent inflammation on the likelihood of acquiring AK (12).

The probability of developing skin tumors is higher due to chronic inflammation, which can occur through two pathways. The first pathway is caused by exposure to UV light and its associated activities. The second pathway is intrinsic and is triggered by genetic changes, including mutations in oncogenes (such as RAS oncogenes), tumor suppressor genes (such as adenomatosis polyposis coli (APC) and TP53), and DNA repair genes (such as MSH-2, MSH-6, and PMS-2). These genetic mutations can lead to cell transformation and the independent proliferation of transformed cells. Moreover, inherent imperfections can potentially cause changes in the immune system, resulting in the generation of substances that cause inflammation and contribute to the development of an inflammatory environment within tumors. Unfortunately, this can also accelerate the progression of the disease (12). Additionally, research has found that *Staphylococcus aureus* (*S. aureus*) could potentially be involved in the onset of AK and the progression from AK to SCC by inducing chronic inflammation. This inflammatory response may involve the production of nitric oxide and cytokines, which contribute to the process of carcinogenesis (25). Moreover, it has been demonstrated that the staphylococcal alpha-toxin can stimulate various cytokines and NF-kB. This provides additional evidence to support the hypothesis that *S. aureus* plays a causative role in the initiation of AK and its progression to SCC (26).

## 4 Oxidative stress

Increasing evidence suggests that oxidative stress is a crucial factor in the formation of skin cancer due to sunlight exposure (27). Reactive nitrogen and oxygen species (RNS and ROS) are produced during various pathological processes, such as DNA damage and lipid oxidation, and are considered significant contributors to the development of tumors in AK and other related conditions (1). Oxidative stress, caused by a weakened antioxidant defense system, plays a role in skin cancer-related aging and cancer formation (28). Different types of tumors generate large amounts of ROS, both inside and outside cells. The *in vivo* generation of reactive ROS can promote aggressive cancer cells, hinder anti-proteases, damage nearby tissues, and encourage tumor heterogeneity, invasion, and metastasis. As a result, malignant tumors maintain higher basal levels of reactive oxygen species compared to normal cells, perpetuating a harmful cycle. It is worth noting that while elevated levels of ROS may lead to oxidative stress and cellular demise, reduced levels of superoxide and H_2_O_2_ can facilitate the G1 → S cell cycle progression in various cellular models. The pathophysiological implications of extracellular ROS should also be considered. Superoxide dismutases (SODs) have been reported to play a crucial role as the primary defense mechanism against injury caused by ROS. These enzymes facilitate the dismutation of the superoxide anion free radical (O_2_-) by catalyzing its conversion into molecular oxygen and H_2_O_2_. This enzymatic action effectively reduces the levels of O_2_-, mitigating cellular damage associated with excessive concentrations of this radical (29). The malignant transformation of AK is highly correlated with an increased level of oxidative status and a significant quantity of ROS (Table 2).

**Table 2:** Epidemiological Literature on AK and cSCC: References from the Past Decade.

Titles	Authors	Journal	DOI	Pub_Date
Impact of COVID-19 Pandemic on Cutaneous Squamous Cell Carcinoma: A Single-Centre Study of Epidemiologic, Clinic and Histopathological Factors	Díaz-Calvillo P et al.	Actas Dermosifiliogr	10.1016/j.ad.2024.01.004	2024
Impact of COVID-19 Pandemic on Cutaneous Squamous Cell Carcinoma: A Single-Centre Study of Epidemiologic, Clinic and Histopathological Factors	Díaz-Calvillo P et al.	Actas Dermosifiliogr	10.1016/j.ad.2023.10.003	2023
The Global Epidemiology of Actinic Keratosis in the General Population: A Systematic Review and Meta-Analysis	George CD; et al.	Br J Dermatol	10.1093/bjd/ljad371	2023
Comparison of the clinical characteristics of benign and malignant eyelid lesions: an analysis of 1423 eyelid lesions, compared between ophthalmology department and plastics department	Levinkron O; et al.	Graefes Arch Clin Exp Ophthalmol	10.1007/s00417-023-06244-5	2023
Alcohol and Health Outcomes: An Umbrella Review of Meta-Analyses Base on Prospective Cohort Studies	Zhong L; et al.	Front Public Health	10.3389/fpubh.2022.859947	2022
Validation of actinic keratosis diagnosis and treatment codes among veterans living with HIV	Supapannachart KJ; et al.	Pharmacoepidemiol Drug Saf	10.1002/pds.5430	2022
Incidence and Prevalence of Skin Cancers in South Korea from 2008 to 2016: A Nation-Wide Population Based Study	Park K; et al.	Ann Dermatol	10.5021/ad.2022.34.2.105	2022
Incidence of Multiple vs. First Cutaneous Squamous Cell Carcinoma on a Nationwide Scale and Estimation of Future Incidences of Cutaneous Squamous Cell Carcinoma	Tokez S; et al.	JAMA Dermatol	10.1001/jamadermatol.2020.3677	2020
Nationwide Incidence of Metastatic Cutaneous Squamous Cell Carcinoma in England	Venables ZC; et al.	JAMA Dermatol	10.1001/jamadermatol.2018.4219	2019
Association of Vitamin A Intake With Cutaneous Squamous Cell Carcinoma Risk in the United States	Kim J; et al.	JAMA Dermatol	10.1001/jamadermatol.2019.1937	2019
Prognostic factors for parotid metastasis of cutaneous squamous cell carcinoma of the head and neck	Bobin C; et al.	Eur Ann Otorhinolaryngol Head Neck Dis	10.1016/j.anorl.2017.09.006	2018
Current perspective on actinic keratosis: a review	Siegel J; et al.	Br J Dermatol	10.1111/bjd.14852.	2017
Incidence, Mortality, and Trends of Nonmelanoma Skin Cancer in Germany	Leiter U; et al.	J Invest Dermatol	10.1016/j.jid.2017.04.020.	2017
Human polyomaviruses and incidence of cutaneous squamous cell carcinoma in the New Hampshire skin cancer study	Gossai A; et al.	Cancer Med	10.1002/cam4.674	2016
Aspirin and nonsteroidal anti-inflammatory drugs can prevent cutaneous squamous cell carcinoma: a systematic review and meta-analysis	Muranushi C; et al.	J Invest Dermatol	10.1038/jid.2014.531	2015
Epidemiology of actinic keratoses	Green AC	Curr Probl Dermatol	10.1159/000366525.	2015
Clinical characteristics of patients with cutaneous melanoma according to variants in the melanocortin 1 receptor gene	Pe?a-Vilabelda MM; et al.	Actas Dermosifiliogr	10.1016/j.ad.2013.10.001	2014
Cutaneous squamous cell carcinoma and human papillomavirus: is there an association?	Aldabagh B; et al.	Dermatol Surg	10.1111/j.1524-4725.2012.02558.x	2013
Sunscreen use on the dorsal hands at the beach	Warren DB; et al.	J Skin Cancer	10.1155/2013/269583	2013
The relevance of the vitamin D endocrine system (VDES) for tumorigenesis, prevention, and treatment of non-melanoma skin cancer (NMSC): Present concepts and future perspectives	Reichrath J; et al.	Dermatoendocrinol	10.4161/derm.24156	2013
Cutaneous squamous cell carcinoma: estimated incidence of disease, nodal metastasis, and deaths from disease in the United States, 2012	Pritesh S; et al.	J Am Acad Dermatol	10.1016/j.jaad.2012.11.037	2013
The natural history of actinic keratosis: a systematic review	R N Werner; et al.	Br J Dermatol	10.1111/bjd.12420	2013
Smoking and the risk of nonmelanoma skin cancer: systematic review and meta-analysis	Leonardi-Bee J; et al.	Arch Dermatol	10.1001/archdermatol.2012.1374	2012
Epidemiologic study of skin diseases among immigrants in Alicante, Spain	Albares MP; et al.	Actas Dermosifiliogr	10.1016/j.ad.2011.07.008	2012
Supplement use and risk of cutaneous squamous cell carcinoma	Asgari MM; et al.	J Am Acad Dermatol	10.1016/j.jaad.2010.09.009	2011
Association of tea consumption and cutaneous squamous cell carcinoma	Asgari MM; et al.	Nutr Cancer	10.1080/01635581.2011.523496	2011
Potential risk factors for cutaneous squamous cell carcinoma include oral contraceptives: results of a nested case–control study	Asgari MM; et al.	Int J Environ Res Public Health	10.3390/ijerph7020427	2010
Occupational exposure to non-artificial UV-light and non-melanocytic skin cancer - a systematic review concerning a new occupational disease	Schmitt J; et al.	J Dtsch Dermatol Ges	10.1111/j.1610-0387.2009.07260.x	2010
Detection of human papillomavirus DNA in cutaneous squamous cell carcinoma among immunocompetent individuals	Asgari MM; et al.	J Invest Dermatol	10.1038/sj.jid.5701227	2008
Guidelines for the management of squamous cell carcinoma in organ transplant recipients	Stasko T; et al.	Dermatol Surg	10.1111/j.1524-4725.2004.30150.x	2004
Presence of human papillomavirus DNA in plucked eyebrow hairs is associated with a history of cutaneous squamous cell carcinoma	Struijk L; et al.	J Invest Dermatol	10.1046/j.1523-1747.2003.12632.x	2003

## 5 Mutagenesis

AK shares similarities with cSCC at the genomic level, exhibiting mutations in 44 driver genes. These genes include TP53, NOTCH1, NOTCH2, FAT1, and KMT2C, among which TP53 is the most commonly mutated gene (30). Other genes that are mutated include the ras genes, c-myc proto-oncogenes, p16INK4a tumor suppressor genes, and genes associated with telomerase activity. However, due to the complexity of these genetic alterations, only a few of them have been identified in current research. The following genes are some of the typical genes among the mutated gene family.

### 5.1 TP53

Skin cancer is frequently associated with genetic abnormalities in the TP53 gene, such as AK (31, 32). TP53 mutations are found in more than 50% of human cancers, including keratinocytic skin cancers. These mutations usually manifest as a CC- > TT base change and are thought to arise during the early stages of long-term sun-induced skin cancer development. To indirectly indicate mutations, TP53 expression has been detected through immunohistochemical methods in various studies (33, 34). Khorshid et al. more than 50% of tumor cells expressed TP53, which was associated with mutations (34). Karagece found TP53 expression in all AK cases, including non-dysplastic areas on H&E slides. The collective findings suggest that TP53 plays a critical role in the early stages of skin cancer development, which is likely triggered by prolonged exposure to UV radiation (35).

### 5.2 MYC

The MYC gene family, consisting of MYC (also known as C-Myc), MYCN (N-Myc), and MYCL1 (LMYC), plays a significant role in promoting epidermal differentiation, cell proliferation, apoptosis, and the development of specific human cancers. One common cytogenetic abnormality observed in the progression from AK to cSCC is the amplification of the MYC gene, which is located in the 8q24 chromosome band. Multiple lines of evidence suggest that MYC may contribute to the lack of differentiation and accelerate the progression from low-grade AK to advanced stages of cSCC. Mutations in the MYC gene that affect DNA replication can lead to a mutator phenotype, triggering a process that enhances proliferation advantage (31).

In AK, MYC amplification may lead to further genomic rearrangements. As a driver gene, MYC can contribute to genomic instability (32). MYC can play a critical role in the transition from a benign pre-cancerous lesion to its malignant form when it carries genetic abnormalities. MYC numerical abnormalities are more common in advanced and undifferentiated stages of the disease, suggesting its involvement in the development of a more aggressive phenotype (31).

### 5.3 TSG

Emerging evidence indicates that the Tumor Suppressor Gene (TSG) plays a vital role in various cellular processes, such as DNA damage repair, cell division inhibition, apoptosis induction, and metastasis suppression. The TSG family encompasses several members, including TP53, p16, p14, APC, MSH2, among others. The inactivation or loss of TSG function is a crucial factor in promoting tumor development (36, 37). Previous studies have suggested that a single copy of TSG can regulate cell growth, while complete inactivation or loss of both alleles is required for tumor formation (17, 36). Additionally, recent findings propose that tumorigenesis is also influenced by the functional deactivation of TSGs through cellular mechanisms such as transcriptional regulation, abnormal cellular localization, and proteasomal degradation (31).

The significance of TSG in the development of skin cancer through exposure to UV radiation has been well-documented. This is evident from the high prevalence of TP53 mutations observed in sun-exposed skin over prolonged periods, as well as in AK and cSCC. Additionally, the disruption of the TSG cluster comprising p14ARF, p15INK4b, and p16INK4a on chromosome 9p21 has been found to promote carcinogenesis. In addition, the skin surrounding AK lesions, which is exposed to sunlight and appears healthy in terms of morphology, showed changes in the expression of p14ARF, p15INK4b, p16INK4a, and TP53 mutations, as documented in Kanellou et al. (31).

### 5.4 The genes IRF4, MC1R and TYR

Mutations in the IRF4 gene, located on chromosome 6p25.3, have a significant impact on melanin synthesis and the host immune response. Down-regulation of IRF4 leads to reduced expression of TYR, a key enzyme in melanin production. Moreover, it adversely affects the toll-like receptor signaling pathway, which is responsible for triggering adaptive immunity. This suggests that a decrease in IRF4 expression may increase vulnerability to AK by weakening the body’s ability to combat abnormal keratinocytes and melanocytes. MC1R, located on chromosome 16q24.3, is a crucial pigmentation gene that regulates eumelanin synthesis by encoding the melanocortin 1 receptor. The rs1805008(T) SNP limits MC1R’s ability to bind with its ligand, resulting in limited melanin synthesis. Melanocytes with loss of function MC1R exhibit reduced DNA repair function after UV exposure, a known cause of skin cancer. This likely contributes to the effects of MC1R on AK, which are independent of pigmentation. TYR, located on chromosome 11q14.3, is responsible for producing the essential enzyme tyrosinase, which plays a crucial role in melanin synthesis. Genetic variations in the TYR gene can lead to reduced enzyme activity, resulting in a lightly pigmented appearance. The risk of developing AK may be higher due to a specific type of genetic variation in the TYR gene, which weakens the immune response to melanocytes. This genetic variation is strongly associated with the aforementioned lightly pigmented phenotype and is identified by the rs1393350(A) single nucleotide polymorphism.

A recent investigation has revealed that the genes IRF4, MC1R, and TYR may have multiple effects, impacting both pigmentation and oncogenic functions. This dual impact may increase the risk of AK. The study also discovered a significant correlation between SNPs and the genes IRF4 locus, SLC45A2, HERC2, MC1R, and TYR. AK is significantly influenced by independent risk factors such as sex, age, and the genes IRF4, MC1R, and TYR. Age and gender were responsible for the majority of AK variance (15%), while the three significant SNPs IRF4, MC1R, and TYR collectively accounted for 2.6%. These findings align with those commonly observed in genome-wide association studies of complex human systems (38–40).

Single-cell RNA sequencing (scRNA-seq) technology provides a powerful method to investigate changes in gene expression at the individual cell level. In a recent scRNA-seq study, researchers identified a group of important candidate genes that may be associated with the development and progression of AK. The study revealed a significant increase in the expression of acetaldehyde dehydrogenase 3A1 (ALDH3A1) and insulin-like growth factor binding protein 2 (IGFBP2) in AK tissues, specifically in epidermal keratinocytes. Interestingly, neither Squamous Cell Carcinoma *in situ* (SCCIS) nor cSCC showed a significant upregulation of ALDH3A1 and IGFBP2, and ALDH3A1 was even found to be downregulated in cSCC. This suggests that ALDH3A1 and IGFBP2 play a distinct role in skin precancerous lesions. The increased expression of ALDH3A1 and IGFBP2, particularly in basal cells, is likely to contribute to the development of AK and may act as key driver genes in the transition from photoaged skin to AK (41).

## 6 Immunosuppression

The immune cells responsible for suppressing the immune response, known as T regulatory cells (Tregs), play a crucial role in the progression of tumors by inhibiting the immune system’s ability to fight tumor cells. The expression of the Foxp3 transcription factor by these cells is highly correlated with the transition from AK to cSCC. Specifically, Tregs producing interleukin-10 (IL-10) and transforming growth factor-beta (TGF-β) hinder the activation of CD4 T cells and dendritic cells, promote growth, and produce various cytokines.

The initial events in UV-induced immunosuppression include the release of platelet-activating factor (PAF) and the conversion of the photoreceptor trans-urocanic acid (tUCA) to the immunosuppressive cis-urocanic acid (cUCA). During UV-induced oxidative stress, PAF receptors activate cytokine transcripts by generating PAF, a phospholipid. PAF and cUCA not only regulate immunosuppression but also impact DNA damage by inhibiting nucleotide excision repair and facilitating the creation of 8-oxo-deoxyguanosine. Additionally, PAF and cUCA promote the production of reactive oxygen species (ROS), which links genetic damage, DNA repair, and immunosuppression.

To summarize, the development of cSCC from AK is highly associated with the rise in Tregs, whereas the release of PAF and the conversion of tUCA to cUCA are two initial occurrences in UV-induced immunosuppression (Table 3).

**Table 3:** Mechanistic Insights into AK Progression to cSCC: References from the Past Decade.

Title	Authors	Journal	DOI	Pub_Date
Single-cell sequencing highlights heterogeneity and malignant progression in actinic keratosis and cutaneous squamous cell carcinoma	Zou DD; et al.	Elife	10.7554/eLife.85270	2023
Targeting *Staphylococcus aureus* dominated skin dysbiosis in actinic keratosis to prevent the onset of cutaneous squamous cell carcinoma: Outlook for future therapies?	Bromfield JI; et al.	Front Oncol	10.3389/fonc.2023.1091379	2023
Driver gene combinations dictate cutaneous squamous cell carcinoma disease continuum progression	Bailey P; et al.	Nat Commun	10.1038/s41467-023-40822-9	2023
Genetic Studies of Actinic Keratosis Development: Where Are We Now?	Lee YB; et al.	Ann Dermatol	10.5021/ad.23.072	2023
Non-Melanoma Skin Cancer and Vitamin D: The Lost Sunlight “Paradox and the Oxidative Stress Explanation”	Karampinis E; et al.	Antioxidants (Basel)	10.3390/antiox12051107	2023
Significant Biomarkers Identification Associated with Cutaneous Squamous Cell Carcinoma Progression	Qiu CG; et al.	Int J Gen Med	10.2147/IJGM.S357022	2022
Skin Cancer-Associated *S. aureus* Strains Can Induce DNA Damage in Human Keratinocytes by Downregulating DNA Repair and Promoting Oxidative Stress	Krueger A; et al.	Cancers (Basel)	10.3390/cancers14092143	2022
Inhibition of Cell Proliferation and Cell Viability by Sinecatechins in Cutaneous SCC Cells Is Related to an Imbalance of ROS and Loss of Mitochondrial Membrane Potential	Zhu J; et al.	Antioxidants (Basel)	10.3390/antiox11071416	2022
Cutaneous Squamous Cell Carcinoma: From Pathophysiology to Novel Therapeutic Approaches	Fania L; et al.	Biomedicines	10.3390/biomedicines9020171	2021
Telomeres and Telomerase in Cutaneous Squamous Cell Carcinoma	Ventura A; et al.	Int J Mol Sci	10.3390/ijms20061333	2019
Neoantigen Fitness Model Predicts Lower Immune Recognition of Cutaneous Squamous Cell Carcinomas Than Actinic Keratoses	Borden ES; et al.	Front Immunol	10.3389/fimmu.2019.02799	2019
The Role of Human Papillomaviruses and Polyomaviruses in BRAF-Inhibitor Induced Cutaneous Squamous Cell Carcinoma and Benign Squamoproliferative Lesions	Purdie KJ; et al.	Front Microbiol	10.3389/fmicb.2018.01806	2018
Immune consequences induced by photodynamic therapy in non-melanoma skin cancers: a review	Yu X; et al.	Environ Sci Pollut Res Int	10.1007/s11356-018-2426-z	2018
A review of BF-200 ALA for the photodynamic treatment of mild-to-moderate actinic keratosis	Reinhold U	Future Oncol	10.2217/fon-2017-0247	2017
MiR-204 silencing in intraepithelial to invasive cutaneous squamous cell carcinoma progression	Toll A; et al.	Mol Cancer	10.1186/s12943-016-0537-z	2016
Gene expression profiling of the leading edge of cutaneous squamous cell carcinoma: IL-24-driven MMP-7	Mitsui H; et al.	J Invest Dermatol	10.1038/jid.2013.494	2014
The role of apoptosis in therapy and prophylaxis of epithelial tumors by nonsteroidal anti-inflammatory drugs (NSAIDs)	Fecker LF; et al.	Br J Dermatol	10.1111/j.1365-2133.2007.07856.x	2007
Analysis of promoter hypermethylation of death-associated protein kinase and p16 tumor suppressor genes in actinic keratoses and squamous cell carcinomas of the skin	Tyler LN; et al.	Mod Pathol	10.1097/01.MP.0000077516.90063.7D	2003

## 7 Impaired apoptosis

Apoptosis is crucial in regulating skin development, homeostasis, and carcinogenesis by balancing epidermal proliferation and removing mutated or potentially cancerous cells. Exposure to UV radiation can lead to the death of skin cells and the development of cancerous growths, as stated in reference (49). Furthermore, UV radiation can cause damage to the DNA of keratinocytes, leading to harmful effects that are not yet fully understood in the process of apoptosis. This is due to the appearance of molecules that either promote or hinder apoptosis (16). The processes described are heavily influenced by the TP53 gene, which functions as a tumor suppressor. TP53 plays a crucial role in activating apoptosis and facilitating cell cycle arrest. Activation of TP53-related genes leads to delayed cell cycle progression, DNA repair, and apoptosis. It also initiates mechanisms for the removal of DNA damage in response to UV-induced damage (49). As mentioned before, the TP53 molecule that encourages programmed cell death is heavily involved in the development of skin cancer and also hinders apoptosis in cells that have DNA damage. Studies suggest that other molecules, such as Human TNF-related apoptosis inducing ligand (TRAIL) and Fas-ligand (FasL), which promote apoptosis, can bypass the immune system (16).

Keratinocytes trigger the process of apoptosis through intrinsic and extrinsic pathways that are regulated by various factors such as MAPKs, JNK, p38, and p53. These factors may be influenced by both environmental and constitutional factors. Apoptosis resistance may occur if there is any deregulation in the critical steps of apoptotic pathways. The deregulation of proteins like Bcl-2, death receptors, and death ligands is often caused by processes such as TP53 inactivation, EGFR overexpression, COX-2 overexpression, and MAPKs overexpression (43).

## 8 HPV infection

In recent years, there has been growing evidence that HPV plays a significant role in the development of AK and cSCC, along with chronic UV irradiation, immunosuppression, and genetic predispositions. A cross-sectional investigation using skin swabs found a correlation between the presence of AK and HPV species 1 and 2 from the Betapapillomavirus genus. In fact, individuals with AK or cSCC, or AK alone, had a higher number of HPV types per sample compared to healthy participants (44). Besides, four novel human betapapillomaviruses of species 2 designated HPV-107, −110 and − 111, and FA75[KI88-03], preferentially found in AK (45). The selective detection of HPV DNA at sites exposed to sunlight could stem from enhanced promoter activity following UV irradiation, coupled with a reduction in apoptosis (46). In particular, the suppression of apoptosis in response to UV-induced damage by the E6 protein from various cutaneous HPV types might significantly contribute to giving genetically damaged keratinocytes a survival edge, leading to the development of AK and cSCC (47). Epidermodysplasia verruciformis-associated HPVs (EV-HPVs) might also play a crucial role in the emergence of AK, as indicated by serological studies, and are implicated in the pathogenesis of SCC (48). In EV-associated cSCCs, a variety of betaHPV types, notably HPV5 and HPV8, are identified. These types are also associated with the onset of actinic keratoses and cSCC in individuals from the general population (49–52). In addition, Bolatti et al. observed a greater prevalence of HPV and higher viral loads in AK compared to cSCC. They also identified a higher prevalence of gammaHPV in AK when compared to betaHPV and alphaHPV types. As a result, it appears challenging to specifically designate high-risk cutaneous HPV types, suggesting that multiple cutaneous HPV types may contribute to tumorigenesis (53). Interestingly, a case study employing the off-label use of the 9-valent HPV vaccine for the management of AK demonstrated regression of AK lesions starting within months of the initial injection. This resulted in the clearance of thousands of lesions even before completing the entire vaccination protocol (54).

## 9 Summary and perspectives on AK studies

There are three possible outcomes for AK: spontaneous disappearance, persistence, or progression to invasive cSCC (11, 14, 55). However, accurately predicting the development of AK lesions is challenging due to current limitations in diagnosis (2). The natural history of AK typically involves high turnover rates, with many lesions developing, regressing, and recurring over time (14). Research indicates that thicker AK lesions have a higher likelihood of progressing to cSCC (56). Several risk factors contribute to this progression. The most significant constitutional risk factors for AK include old age, male gender, fair skin, immunosuppression, and a previous history of AK. Additionally, chronic sun exposure is the most significant environmental factor contributing to AK (13, 57–60). Individuals with HPV infection or chronic lymphocytic leukemia who have undergone solid-organ transplantation are at a greater risk of developing cSCC compared to the general population (61–66). In summary, AK can progress to cSCC and serves as a pre-cancerous lesion (49). The development of cSCC involves molecular pathways, including genomic instability caused by TP53 mutations induced by UV radiation (67).

Compared to other types of solid tumors, the development of cSCC involves multiple genetic mutations, which may have potential therapeutic implications (68). Additional genetic alterations occur in tumor suppressor genes such as CDKN2A and NOTCH (69), as well as in oncogenes such as RAS (68). The accumulation of these gene mutations activates various signaling pathways, including NF-kB, MAPK, and PI3K/AKT/mTOR pathways (70, 71), leading to the overexpression of the epidermal growth factor receptor (EGFR). A recent study found no significant correlation between numerical gains in EGFR and tumor depth or size. However, the study suggests that EGFR numerical aberrations occur during the early stages of cancer development. Currently, there is no available literature assessing the predictive role of EGFR cytogenetic aberrations in the treatment of metastatic or recurrent SCC with tyrosine kinase inhibitors. Furthermore, the effectiveness of anti-EGFR drugs in treating AK remains unexplored. Nevertheless, reports indicate that patients undergoing treatment with erlotinib experience inflammatory flare-up reactions resulting in partial destruction of AK. In summary, a significant proportion of *in situ* SCC already exhibit EGFR numerical gains, but these alterations do not appear to contribute to the progression from low-grade SCC to more aggressive phenotypes (71). Studies have revealed that several signaling pathways, which are activated in cSCC, exhibit pre-existing activation in AK, thereby supporting AK as precursor lesions of cSCC.

Through comprehensive genome-wide SNP microarray and expression microarray analyses, pathways such as NF-kB1 and the tumor necrosis factor pathways have been identified. It is noteworthy that both NF-kB1 and the tumor necrosis factor pathways are classic proinflammatory signaling pathways. Another investigation sheds light on the involvement of the MAPK pathway and apoptosis-related genes in the pathogenesis of cSCC and AK. These findings underscore the participation of pathways related to cell cycle regulation, apoptosis, inflammation, and epidermal differentiation in the development and progression of cSCC from AK (15, 19). A recent study discovered dysregulation of TGFβ signaling that varies depending on the progression stage, ranging from normal skin to AK to cSCC. One group of TGFβ-associated genes consistently showed increased activity throughout the progression, while another group exhibited decreased activity. These findings indicate the potential involvement of TGFβ signaling in the transition from AK to cSCC (20). The study of signaling pathways can offer potential targets for future treatments of AK and cSCC. Furthermore, it has been observed that epigenetic alterations may occur during the progression of AK and cSCC. Several studies have investigated the use of DNA methylation arrays to assess AK (21–35). It has been proposed that during the transition from normal skin to AK and cSCC, there may be an increase in E-cadherin promoter hypermethylation (35). Furthermore, the malignant potential of AK has been highlighted by observing AK methylomes exhibiting typical cancer-related characteristics, including CpG island promoter hypermethylation and hypomethylation of lamina-associated domains (21). DNA methylation signature could discriminate different stages of disease ranging from premalignant AK to low-risk invasive and high-risk non-metastatic and metastatic CSCC in the future.

Genetic alterations, such as TP53 mutations, ras gene mutations, c-myc proto-oncogene mutations, p16INK4a tumor suppressor gene mutations, and telomerase activity, are closely associated with the development of AK. The progression of AK lesions is difficult to predict, but thicker lesions are more likely to progress into cSCC (3). Environmental and constitutional risk factors for AK include chronic sun exposure, advanced age, male gender, fair skin, immunosuppression, previous history of AK, and HPV infection. EGFR overexpression is linked to various pathways, including NF-kB, MAPK, and PI3K/AKT/mTOR. These pathways can be activated by UV-induced TP53 mutations, CDKN2A and NOTCH alterations, and RAS mutations (72–78).

This review has certain limitations that should be considered. Firstly, the current research on the pathogenesis of AK is not comprehensive enough. Secondly, our findings were drawn from existing literature and evaluations, underscoring the imperative for additional enhancements in assessment methods within the field to attain a more comprehensive understanding of the pathogenesis of AK. However, we hypothesize that AK has the potential to progress to cSCC, and timely intervention in the signaling pathways could lead to successful treatment. It is crucial to protect the skin from sunburn damage, as UV radiation is the primary cause of AK. Technological advancements have facilitated the identification of more genes associated with AK, offering potential targets for treatment. We speculate that with research providing deeper understanding of AK pathogenesis, it could be diagnosed more accurately in the future and treated with more effective medications.

## 10 Conclusion

In conclusion, AK is a skin disorder that is increasingly prevalent and has the potential to progress to cSCC. The development of AK involves various intricate mechanisms, which offer potential avenues for treatment. Timely diagnosis, treatment, and prevention of AK are of utmost importance. Further research is needed to enhance our comprehensive understanding of this disease.

## Author contributions

ZW: Writing – original draft, Writing – review & editing. XW: Conceptualization, Writing – original draft. YS: Data curation, Writing – original draft. SW: Formal analysis, Writing – review & editing. YD: Software, Writing – original draft. GY: Methodology, Project administration, Writing – original draft. JC: Writing – review & editing.
